# Biotechnology and Biomaterial-Based Therapeutic Strategies for Age-Related Macular Degeneration. Part II: Cell and Tissue Engineering Therapies

**DOI:** 10.3389/fbioe.2020.588014

**Published:** 2020-12-10

**Authors:** Nahla Jemni-Damer, Atocha Guedan-Duran, María Fuentes-Andion, Nora Serrano-Bengoechea, Nuria Alfageme-Lopez, Félix Armada-Maresca, Gustavo V. Guinea, José Perez-Rigueiro, Francisco Rojo, Daniel Gonzalez-Nieto, David L. Kaplan, Fivos Panetsos

**Affiliations:** ^1^Neuro-computing and Neuro-robotics Research Group, Complutense University of Madrid, Madrid, Spain; ^2^Innovation Group, Institute for Health Research San Carlos Clinical Hospital, Madrid, Spain; ^3^Department of Biomedical Engineering, Tufts University, Medford, MA, United States; ^4^Silk Biomed SL, Madrid, Spain; ^5^Ophthalmology Service, La Paz University Hospital, Madrid, Spain; ^6^Center for Biomedical Technology, Universidad Politécnica de Madrid, Pozuelo de Alarcon, Spain; ^7^Department of Material Science, Civil Engineering Superior School, Universidad Politécnica de Madrid, Madrid, Spain; ^8^Biomedical Research Networking Center in Bioengineering, Biomaterials and Nanomedicine, Madrid, Spain

**Keywords:** retinal pigment epithelium, Bruch’s membrane, photoreceptors, biomaterials, cell therapy, tissue engineering, biotechnology, cell replacement

## Abstract

Age-related Macular Degeneration (AMD) is an up-to-date untreatable chronic neurodegenerative eye disease of multifactorial origin, and the main causes of blindness in over 65 y.o. people. It is characterized by a slow progression and the presence of a multitude of factors, highlighting those related to diet, genetic heritage and environmental conditions, present throughout each of the stages of the illness. Current therapeutic approaches, mainly consisting on intraocular drug delivery, are only used for symptoms relief and/or to decelerate the progression of the disease. Furthermore, they are overly simplistic and ignore the complexity of the disease and the enormous differences in the symptomatology between patients. Due to the wide impact of the AMD and the up-to-date absence of clinical solutions, Due to the wide impact of the AMD and the up-to-date absence of clinical solutions, different treatment options have to be considered. Cell therapy is a very promising alternative to drug-based approaches for AMD treatment. Cells delivered to the affected tissue as a suspension have shown poor retention and low survival rate. A solution to these inconveniences has been the encapsulation of these cells on biomaterials, which contrive to their protection, gives them support, and favor their retention of the desired area. We offer a two-papers critical review of the available and under development AMD therapeutic approaches, from a biomaterials and biotechnological point of view. We highlight benefits and limitations and we forecast forthcoming alternatives based on novel biomaterials and biotechnology methods. In this second part we review the preclinical and clinical cell-replacement approaches aiming at the development of efficient AMD-therapies, the employed cell types, as well as the cell-encapsulation and cell-implant systems. We discuss their advantages and disadvantages and how they could improve the survival and integration of the implanted cells.

## Introduction

### Age-Related Macular Degeneration

Age-Related Macular Degeneration (AMD) is a multifactorial degenerative eye disease, estimated to affect nearly 290 million people by 2040 (Wong et al., 2014). It is characterized by the deterioration of the central retinal area in the elderly population leading to vision deterioration and even blindness [see Therapeutic Strategies for Age-Related Macular Degeneration Part I and Part II, also (Hoon et al., 2014; Wong et al., 2014)].

**FIGURE 1:**
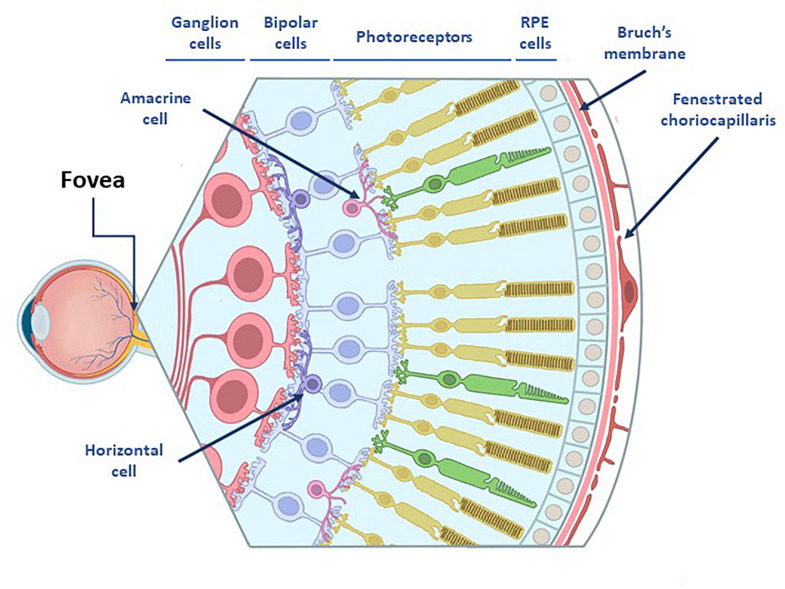
Representation of the eye anatomy. The retina consists on 3 nuclear layers: (I) tanner nuclear layer which consists of ganglion cells bodies whose axons will form the optic nerve (II) the middle nuclear layer which are located the bipolar cell bodies and (III) the outer nuclear layer which includes photoreceptors, those cells lies over three non-neural layers: Choroid, Bruch’s membrane (BrM) and retinal pigment epithelium (RPE) cells. In between the nuclear cell layers are two plexiform layers which contain amacrine and horizontal cells whose function is the modulation of the activity in the bipolar and ganglion cells via lateral inhibition.

The retina is the inner component of the eyeball (Figure 1). It is a 10-layers complex structure, where a several types of neural cells, photoreceptors, bipolar, horizontal, amacrine and retinal ganglion cells, are tightly interconnected by means of chemical and electrical synapses to form a network. Visual perception starts at photoreceptors, highly specialized retinal neurons which convert light into electric signals. Retina’s outer sheet, thePhotoreceptors, lies over three non-neural layers: choroid, Bruch’s membrane (BrM) and retinal pigment epithelium (RPE), all of them strongly involved in the development of the AMD (Figure 1; Hoon et al., 2014; Wong et al., 2014). Retina’s inner layer is in contact with the vitreous humor, a non-vascularized transparent gelatinous medium that fills the interior of the eyeball, whose function is to maintain the shape of the eye and smooth the retinal surface to form sharp images.

**FIGURE 2:**
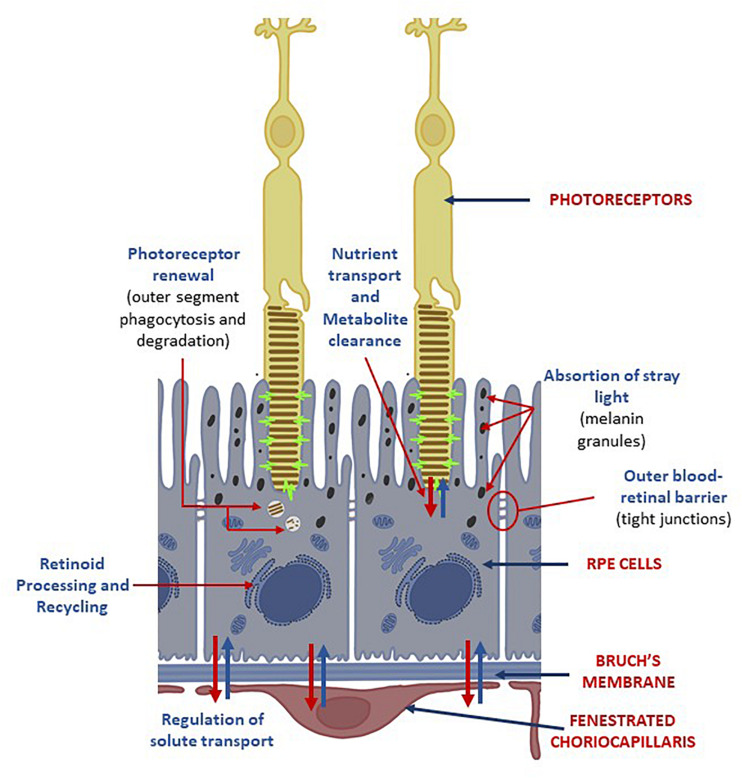
Choroid-RPE-Photoreceptors interactions. RPE cells are located between the light-sensitive outer segments of the photoreceptors and the fenestrated choriocapillaris from the choroid. Its apical membrane faces the subretinal space and it’s specialized to enable interactions between the monolayer of RPE and the outer segments of the photoreceptors. The functions of RPE cells on the neural retina are: (I) absorption of tray light by its abundant melanin granules; (II) retinoid processing and recycling; (III) transport of nutrients and metabolites: (IV) degradation of photoreceptors’ outer segments. Its basolateral membrane is in contact with the Bruch’s membrane. The tight junctions between RPE cells create a selective blood-retinal barrier that regulates the flow of nutrients, metabolic waste products, ions, proteins and water into and out the retina.

The choroid is a vascularized layer that supplies oxygen and nutrients to the whole retina. Additionally, it serves as physical support and it also contributes to the maintenance of the intraocular pressure and the regulation of eye’s temperature (Marmor and Wolfensberger, 1998; Michalska-Małecka et al., 2015). BrM is a 2–5 μm-thick acellular sheet, mainly composed of elastin, collagen I-V, laminin and fibronectin and it is divided into five layers: the choriocapillary basement membrane, the outer collagenous layer, the central band of elastic fibers, the inner collagenous layer and the RPE basement membrane (Figure 2; Hogan et al., 1971; Booij et al., 2010). It gives physical support to the RPE cells and regulates the exchange of molecules, oxygen, nutrients and metabolic residues between the choroid and the RPE (Hogan et al., 1971; Booij et al., 2010). RPE is a monolayer of highly specialized hexagonal cells that are in direct contact with the outer segment of the photoreceptors. It is dedicated to the absorption of scattered light, to the secretion of growth factors, the transport of nutrients from the choroid to the neural cells, the phagocytosis of the outer segment of the photoreceptors and, together with BrM, to the formation of the blood-retinal barrier (Figure 2; Cunha-Vaz, 1997; Campbell and Humphries, 2013). RPE cells play a key role in the maintenance of the visual function and the survival of the photoreceptors (Hoon et al., 2014; Wong et al., 2014) and, similarly to what happens to the neural cells, RPE cells cannot regenerate after birth (Marmor and Wolfensberger, 1998; Bharti et al., 2006; Cunha-Vaz et al., 2011; Jin et al., 2019).

Since choroid, BrM and RPE provide physical support for the neural retina, and since they are in charge for both, the interchange of the metabolic molecules and the correct physiological functioning of the photoreceptors, any degenerative pathology affecting one of these layers, would also impair or even kill the photoreceptors with the consequent visual impairment or even blindness. In AMD, BrM weakens and thickens, thus losing its permeability and diffusive properties (Booij et al., 2010). Low permeability and diffusion capability provoke an accumulation of the lipids and the cellular/metabolic residues that are originated in the RPE and the surrounding tissue, giving rise to drusen formation between RPE and BrM. In turn, these changes cause loss of the integrity of the external blood-retinal barrier as well as death of the RPE cells (Marmor and Wolfensberger, 1998; Booij et al., 2010). Due to RPE extensive interactions with the photoreceptors and because RPE cells are unable to regenerate after birth, RPE dysfunction or dead can cause permanent loss of vision capabilities (Marmor and Wolfensberger, 1998; Bharti et al., 2006; Cunha-Vaz et al., 2011; Jin et al., 2019). When alterations occur in the choroid, they give origin to the most aggressive form of AMD, the wet one (W-AMD): choriocapillar alterations provoke uncontrolled angiogenesis, sub-choroidal neovessels penetrate the subretinal space through existing BrM/RPE defects and destroy the morphology and cellular structure of the retinal tissue (Marmor and Wolfensberger, 1998; Michalska-Małecka et al., 2015).

In the human eye, there are approximately 137 million photoreceptors: 7 million cones and 130 million rods. Cones are concentrated in the center of the eye (in the fovea) and their density decreases radially from the center to the periphery. Rods are concentrated at about 20° from the center (approx. 6 mm from the fovea) and their density also decreases toward the periphery but, slower than the cones. Photoreceptors’ degeneration is irreversible because all cellular regeneration and repair processes are inhibited by the unfavorable molecular conditions that prevail throughout the central nervous system. Among them, the absence or inappropriate concentration of neurotrophic and/or neurotropic factors, the appearance of inflammatory processes, astrocytic reactions and inhibitory molecular signals (myelin binding proteins or excess of neurotransmitters and ions), as well as the presence of myelin-associated factors and/or glial scar (Bray et al., 1987; Aguayo et al., 1991; Sauve and Gaillard, 1995; Liu et al., 2006).

Drugs, molecules and factors’ administration are the prevalent therapeutic options today, none of them being a therapy for the disease. They only contribute to slow down the progression of the pathology, with the aggravating factor that they are effective in only a small percentage of patients (Gehrs et al., 2006; Sabel et al., 2011; Irisvision, 2020). For this reason, in the last decades, serious efforts have been done to develop new, more effective treatments based either on the implant of stem cells that are used as micro-factories producing *in situ* neuroprotective and neuroregenerative biomolecules (Emerich and Thanos, 2008; Augustin et al., 2012; Guerrero-Naranjo et al., 2013; Barar et al., 2016), or on the replacement of damaged cells by autologous or allogeneic cell transplants (Gaillard and Sauvé, 2007; MacLaren and Pearson, 2007; Singh and MacLaren, 2011; Bharti et al., 2014; Singh et al., 2020).

### The Immunoprivileged State of the Eye

The inflammatory response in the eye is regulated by its own immunosuppressive microenvironment, a series of complex regulatory systems which include vasoactive peptides (α-melanocyte-stimulating hormone, a regulator of the adaptive immune response, and calcitonin gene-related peptide), macrophage migration-inhibitory factor, and soluble CD95L (which regulates the innate immune response) (Taylor, 2016). Furthermore, the eye complement system plays an important role in the production of inflammatory cytokines (Goslings et al., 1998). Multiple complement factors have been described in the eye of both, human and mice (Anderson et al., 2010; Luo et al., 2011), which can be regulated by the CD46, CD55, CD59, and Crry proteins, expressed by microglia and RPE cells.

However, at the same time, the eye is one of the few immunoprivileged tissues, characterized by the presence of negative regulators which prevent the activation of local inflammatory processes (Medawar, 1948) and where implants can survive for an extended period of time (Streilein, 2003). The biological role of this immunological privilege is to avoid vision deterioration in case of overreaction of the immune system (Sandhu et al., 2019).

Immunoprivileged conditions are the result of a synergy of physical, molecular and cellular barriers (Taylor, 2016) implemented by inflammation suppressors and down-regulators of the immune system, located either on cell membrane or the extracellular tissue (Taylor and Ng, 2018).

Blood-retina barrier and the indirect draining of the ocular microenvironment by the lymphatic system constitute the physical barriers. Blood-retina barrier is formed by the endothelial junctions of RPE cells which impede immune system cells to leak into the eye (Niederkorn et al., 1981). The indirect draining of the eye eliminates the necessity of a channel to the interior of the eye, which increases the difficulty for the immune system to reach the ocular tissue and enhances the efficiency of the blood-retina barrier. TGF-β2 and other soluble immunomodulatory molecules present in the aqueous humor constitute the molecular barrier, whose role is to attack and neutralize the cells of the immune system and to control inflammation (Taylor, 2016). Three antigen-presenting cell types, microglia, perivascular macrophages and dendritic cells, are believed to form the cellular barrier (Forrester and Xu, 2012): macrophages participate in the maintenance of vascular homeostasis in the retina and are believed to be capable of antigen presentation, although microglia is the primary antigen-presenting cell type of the eye.

Age-related macular degeneration-originated damages of the RPE alter this state of the eye. Cellular and tissue engineering AMD therapy procedures, either injections or surgical implants, also contribute to the worsening of the situation.

## Cell and Tissue Engineering Therapies

The retina, the tissue most severely by AMD is an easy target for cell and tissue engineering therapeutical approaches, because of its location and its small size, which allow easy surgical accessibility and transplantation of smaller amounts of replacement cells compared to other organs. Besides, the availability of a large number of clinical variables for the assessment of the visual function, facilitated the precise evaluation of the effectiveness of novel AMD therapies (Bennett et al., 2019) and fostered the development of several regenerative approaches based on BrM, RPE and photoreceptor cells replacement (Chichagova et al., 2018).

**FIGURE 3:**
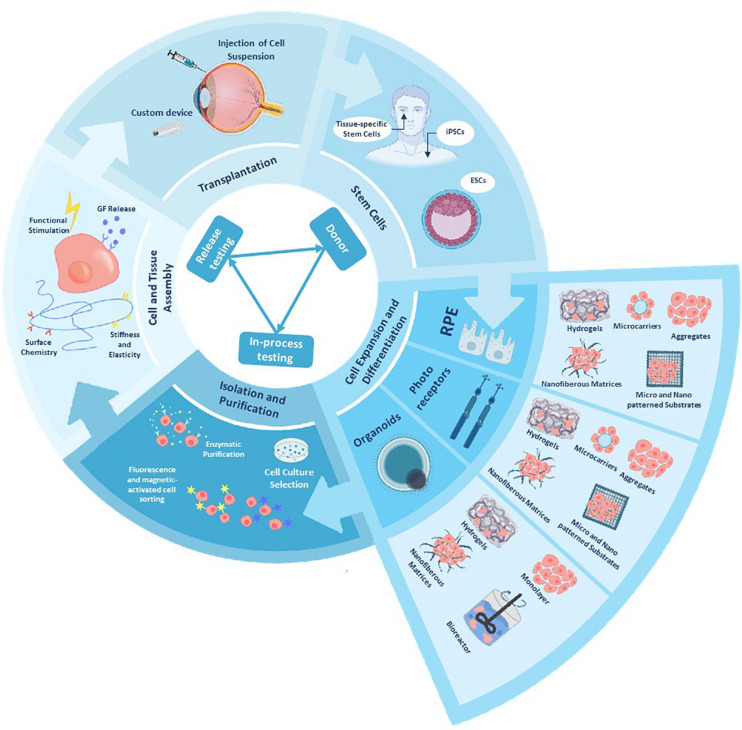
Schematic representation of a tissue bioengineering model, applied to cell and tissue manufacture and delivery. Manipulating and differentiating stem cells into different retinal cells is a challenging with common goals as achieving manufacturing standard and testing for each clinical stage as well achieving an efficient manufacturing and banking. Bioengineering approaches can provide us with scalable 3D cell culture systems that allow differentiation into different retinal cells, biomanufacturer and bioassembly of functional eye tissues and delivery vehicles for an *in vivo* model [after (Stern et al., 2018)].

Biomolecules can be used for therapeutic reasons on a neural tissue, through two, non-mutually exclusive mechanisms. On one side, through the application of neuroprotective and neuroregenerative factors (directly injected to the neural tissue or secreted from implanted stem cells) which diffuse into the host tissue and rescue the degenerating cells. On the other, by implanting healthy cells into the host tissue and replacing the damaged/degenerated cells by the new ones. In the first case, to avoid molecules/cells dispersion into the host tissue, encapsulation can be employed. Porous materials permits the signals of the damaged environment to reach the implanted cells and stimulate the production of neuroprotective biomolecules, as well as to allow the diffusion of the secreted molecules to the damaged tissue (Battler and Leor, 2006; Weiner, 2008; Seiler and Aramant, 2012; Lin et al., 2017; Fernández-García et al., 2018; González-Nieto et al., 2018). However, although neuroprotective factors could protect retinal cells before apoptosis occurrence, their application is not a viable solution for many patients with advanced retinal pathologies. In the second case, cell replacement strategies get advantage of the encapsulation of therapeutic cells in biomaterial formats with an optimal mesh size to obtain a functional connection of donor cells with host tissue; however, this strategy faces the risk of a higher exposition to inflammatory responses that can be detrimental for the grafted cells as well as the risk of rejection of the allogenic material by the host tissue (Figure 3).

### Cell Sources for Retinal Implants

Cells source is a key component to the success of any cell therapy. We can identify three main sources for retinal implants:

#### Embryonic or Fetal-Derived Stem Cells

They represent an attractive source due to their ability to self-renew and differentiate into any type of cell in the body (pluripotent). However, they also present serious disadvantages, e.g., difficult and expensive cell differentiation and cell expansion protocols, complex bioengineering processes, tumor formation risks, rejection risks, possibility of harboring donor’s genetic defects, possible ethical challenges, etc. (Arnhold et al., 2004; Aoki et al., 2009; Lu et al., 2009; Falkner-Radler et al., 2011; Schwartz et al., 2012; Carr et al., 2013; Gonzalez-Cordero et al., 2013; Jayakody et al., 2015; Lu and Barnstable, 2017).

#### Adult Tissue-Derived Stem Cells

They are a source of autologous multipotent cells. However, these cells could be hard to obtain and they could harbor the genetic cause of the disease (West et al., 2009; Ballios and van der Kooy, 2010; Mead et al., 2013, 2015; Hertz et al., 2014; Park et al., 2017).

#### Induced Pluripotent Stem Cells

They are a relatively new source of stem cells developed by directly reprogramming adult somatic cells to transit to a pluripotent state. The use of iPSCs is exciting because iPSCs can be derived from the patient’s own tissue and are associated with fewer ethical concerns than ESCs. Using a combination of soluble factors, iPSCs can be expanded and differentiated into various types of retinal cells, including rods, cones, and retinal ganglion cells. They present a very low rejection risk, however, they could conserve epigenetic characteristics of the original cells, harbor the disease genes from the donor and lead to tumor formation (Rowland et al., 2012; Kamao et al., 2014; Clegg et al., 2015; Mead et al., 2015; Fields et al., 2016; Rajala and Gardner, 2016; Bhattacharya et al., 2017; Bracha et al., 2017). The positive outcomes of early-phase trials postulate iPSCs as the most promising choice (Table 1; Chichagova et al., 2018).

**TABLE 1:** Cellular types employed in AMD cell therapy.

Cell Type	Source	Advantages	Disadvantages
Autologous ocular tissue [1]	IPE cells, peripheral RPE patch or as suspension, Bruch membrane	Derived from the same embryonic cell line, can be easily collected	Can retain original epigenetic features, harbors donor’s disease genes
Adult TDSC [2]	Bone marrow-derived mSC, adipose-derived cells, neural progenitor SC, umbilical tissue cells, RPE cells, Müller cells, ciliary margin zone SC	Multipotent, no rejection risks, source of factors providing neuroprotection	Hard to harvest, can harbor donor’s disease genes
Fetal or early postnatal retinal progenitor cells [3]	Neuroretina, RPE sheet, combined RPE and retinal sheet, retinal progenitor cells	Feasible source of cell, low immunogenicity and not always rejected	Ethically challenging, likely to be rejected, harbors donor’s disease genes
ESCs [4]	Inner cell mass of blastocyst	Pluripotent, self-renew ability, differentiated to all retinal cell types	Likely to be rejected, harbors donor’s disease genes, risk of tumor formation, ethically challenging
iPSC [5]	Somatic cell (e.g., skin fibroblast) with factor- based reprogramming	Pluripotent, easy to grow, low rejection risk, differentiated to all retinal cell types	Can retain original epigenetic features, harbor donor’s disease genes

*IPE, iris pigment epithelium; RPE, retinal pigment epithelium; TDSC, tissue-derived stem cells; mSC, mesenchymal stem cells; SC, stem cells; ESCs, embryonic- or fetal- derived stem cells; iPSC, induced pluripotent stem cells; PR, photoreceptors. [1] (Crafoord et al., 2002; Falkner-Radler et al., 2011; van Zeeburg et al., 2012); [2] (Salero et al., 2012; Davis et al., 2017); [3] (Seaton et al., 1994; Little et al., 1998; Das et al., 1999); [4] (Schwartz et al., 2012); [5] (Carr et al., 2009).*

### Encapsulation of Biomolecule-Producing Stem Cells

At the present, cell encapsulation represents the preferred therapeutic approach since direct injection of cells has been proven to be suboptimal, due to cell losses from the rapid dispersion into the host tissue, along with glia and host immune system attacks.

#### *In vitro* Studies

In their attempt to create artificial BrM, (McCormick et al., 2020) generated a poly (ethylene terephthalate) (PET) scaffold with poly (lactic acid-co-glycolic acid) (PLGA) or poly (glycolic acid) (PGA) degradable nanoparticles that exhibited a continuous release of a fluorescent dye from PLGA particles for 2 weeks and from PGA particles for 1 day.

For cell encapsulation Bhatt et al. (2019) focused on composites by encapsulating retinal pigment epithelial cell line-19 model (ARPE-19) in PLGA nanoparticles of sunitinib malate, which in turn were included in thermosensitive hydrogels (from methoxy poly (ethylene glycol)-b-copolymers of polycaprolactone (mPEG-PCL)), to increase the residence time of the particles in the vitreous humor. In an *in vitro* study they showed that their device had better drug absorption, increased antiangiogenic potential, and prolonged inhibition of vascular endothelial growth factor (VEGF) activity compared to the free drug solution (Bhatt et al., 2019).

#### Clinical Trials

NT-501, NT-503 and iTrack275 Microcatheter, three products developed for the encapsulation of biomolecule-producing stem cells, are aiming at obtaining Food and Drug Administration (FDA) approval for AMD treatment (Table 2):

**TABLE 2:** Cell encapsulation technologies for solid implantable devices integrating stem cells for *in situ* production of therapeutic biomolecules.

Product	Secretome	Type	Description	TRL	Advantages	Disadvantages	Duration
Renexus NT-501 [1]	CNTF	Polysulfone	Polysulfone scaffold with hRPE cells, intravitreally injected	II/III	No immune response, no risk of implant migration, no serious side effects	Needs surgical removal, no improvement	18 m
NT-503 [2]	VEGFR-Fc	PET membrane	Semipermeable PET membrane containing hRPE cells, intravitreally injected	II	No immune response, no risk of implant migration	Needs surgical removal, no serious side effects	12 m
iTrack275 [3]	Bevacizumab/Stem cells (CNTO 2476)	Microcatheter + optic fiber + pump	Supra-choroidally injected	II	Controlled delivery of drugs and human stem cells	Surgically implanted and removed, no serious side effects	Refillable

*CNTF, ciliary neurotrophic factor; RPE, retinal pigment epithelium; VEGF, vascular endothelium growth factor; VEGFR-Fc, VEGF receptor fragment crystallizable region; PET, polyethylene terephthalate. All devices are non-biodegradable. [1] (Neurotech Pharma) (Sieving et al., 2006; Emerich and Thanos, 2008; Birch et al., 2013); [2] (Neurotech Pharma) (Guerrero-Naranjo et al., 2013; Neurotech, 2020); [3] (Johnson and Johnson/iScience, Inc.) (Ho et al., 2017; U.S. National Library of Medicine, 2020a).*

NT-501 (Renexus, Neurotech Pharma) is a non-biodegradable polysulfone scaffold to encapsulate genetically engineered human RPE cells. The device is injected intravitreally and provides a sustained delivery of ciliary neurotrophic factor for months. The device is sutured into the sclera by a titanium loop to avoid migration risks and requires surgery to get removed. The device is in Phase III clinical trials and it has been effective for up to 18 months providing photoreceptor protection (Tao et al., 2002; Bush et al., 2004; Sieving et al., 2006; Emerich and Thanos, 2008; Kuno et al., 2011; Birch et al., 2013; Bisht et al., 2017; U.S. National Library of Medicine, 2020b, g).

NT-503 (Neurotech Pharma) is a non-biodegradable polyethylene terephthalate semipermeable hollow fiber membrane that encapsulates genetically engineered human RPE cells that synthesize and secrete a VEGF receptor fragment crystallizable region. This device is implanted intravitreally through a peritomy and subsequent 3 mm sclerotomy and it has been shown to maintain therapeutic effects for 12 months in W-AMD and was 20 times more efficient than injections of Ranibizumab. As with NT-501, the device gets fixed to the sclera to avoid migration and also requires surgery to be removed. This device is in Phase II clinical trials (Sieving et al., 2006; Guerrero-Naranjo et al., 2013; Rupenthal, 2017; U.S. National Library of Medicine, 2020u).

iTrack275 Microcatheter (iScience Interventional) is a micro-calibrated pump for supra-choroidal drug/cell delivery, surgically inserted through a non-biodegradable microcatheter that includes an optical fiber illuminator to guide the insertion of the device. This suprachoroidal pump allows an improved supply of drugs, a longer duration and a greater penetration into the tissue due to the proximity to the choroid and the BrM, with the consequent avoidance of blurred vision, changes in the lens and in the vitreous (traction exerted), compared to pharmacological delivery by intravitreal or periocular injection. The device has been used in combination with Bevacizumab in advanced AMD and with cells derived from human umbilical tissue (CNTO 2476) for dry-AMD (D-AMD), showing good safety and efficacy with no serious complications (Kuno et al., 2011; Augustin et al., 2012; Pearce et al., 2015; Bansal et al., 2016; Rupenthal, 2017). Phase I/IIa trials were performed with CNTO 2476 using iTrack275, for the administration of a single subretinal dose. Although, it was associated with a high rate of retinal perforations and retinal detachments, when the cells were “sequestered” by the subretinal space, it was well tolerated and was associated with some improvement in visual acuity. Larger studies are required to confirm these results (Ho et al., 2017; U.S. National Library of Medicine, 2020a).

### *In situ* Cell Replacement

#### BrM-RPE Transplantation

##### Early attempts

Early attempts of AMD tissue reconstruction consisted of either autologous (translocated explants) or allogenic (fetal origin) RPE transplants, to replace the damaged retinal tissue (Steindl and Binder, 2008) by substituting the whole choroid-BrM-RPE complex (Ruysch’s complex) via an RPE-choroid explant or by an RPE layer alone. Despite some promising results on the autologous strategy in a small portion of patients (van Zeeburg et al., 2012) aiming at slowing the progression of the pathology and/or improving visual capability, these therapeutic approaches were abandoned due to a number of serious drawbacks. The most significant problems were the limited availability of autologous tissue and the maintenance of genetic risks to develop the disease. Even though allogenic transplants showed better results, today, RPE transplants with natural membranes have been totally replaced by implants based on biomaterials and eye/stem cells. However, the beneficial effects of these autologous and allogenic transplants served as a proof-of-principle for the subsequent cell therapy strategies (Hynes and Lavik, 2010; Yao et al., 2011; Kim et al., 2012; Tam et al., 2014).

##### *In vitro* and pre-clinical trials

Retinal pigment epithelium degeneration can lead to the development of more advanced AMD stages so, transplants of healthy RPE cells have been considered as candidates for the deceleration of the disease (Ambati and Fowler, 2012). Vision improvement of AMD patients subject to RPE cell transplantation supported this approach (van Zeeburg et al., 2012). The specific requirements for improving physiological functions and fostering the survival of RPE cells, are thickness, flexibility, permeability and biodegradability of the implant, as well as easiness to handle during surgical intervention (Hynes and Lavik, 2010). Artificial BrM needs to be biocompatible while promoting and maintaining a proper RPE phenotype. Good integration with the choroid is also sought. In addition, artificial membranes must be porous, thin (less than 10 μm, mimicking BrM) and mechanically competent to withstand manipulation during surgery. Since damaged BrM can also compromise the success of cell transplantation, several researchers dedicated their efforts to the development of artificial membranes allowing successful transplantation of RPE cells (Srivastava et al., 2011; Hu et al., 2012; Warnke et al., 2013; McHugh et al., 2014; Stanzel et al., 2014; Ilmarinen et al., 2015; Peng et al., 2016; Calejo et al., 2017; Harris et al., 2019; McCormick et al., 2020).

A number of artificial scaffolds seeded with RPE cells, mimicking a healthy BrM-RPE complex, have been investigated. Collagen has been one of the most experimented materials because it is one of the fundamental components of BrM: (Lu et al., 2007) cultured human ARPE-19 cells in 2.4 μm-thick collagen films confirming cell attachment and viability at 25 days and a suitable cell phenotype. Other polymeric substrates have been tested *in vitro* and/or *in vivo* with comparable results: gelatin (Del Priore et al., 2004; Lai, 2013; Rose et al., 2014), poly-methyl-methacrylate (PMMA) (Tao et al., 2007), modified polytetrafluoroethylene (PTFE) (Maminishkis et al., 2016), polyethylene terephthalate (PET) (Kamao et al., 2014; Liu et al., 2014, 2018; Jin et al., 2018), polyester matrix membranes (Liu et al., 2014; Stanzel et al., 2014), poly-caprolactone (PCL) (Redenti et al., 2008; McHugh et al., 2014; Tam et al., 2014), poly(L-lactic acid) blends (PLLA/PLGA) (Giordano et al., 1997; Lu et al., 1998; Hadlock et al., 1999; Warnke et al., 2013), polyethylene glycol di-methacrylate (PEGDMA) (Singh et al., 2001), polydimethylsiloxane (PDMS) (Peng et al., 2016). Silk fibroin was also used as a versatile material for scaffolds since it has a number of functional groups that can react with biomolecules of interest like arginine–glycine–aspartic acid (RGD) (Chirila et al., 2008, 2015; Wenk et al., 2011; Kundu et al., 2013; Xiang et al., 2014; Zhang et al., 2015), all of them in experimental phase.

Synthetic BrMs consisting of PET scaffolds and degradable nanoparticles were developed by McCormick et al. (2020). ARPE-19 cells cultured on these scaffolds were able to form monolayers and were maintained for up to 3 months without any cytotoxic effect. This proof-of-concept showed the potential of this nanoparticle-scaffold system for future RPE transplantation. In another synthetic BrM, composed of collagen type I and PLGA, cultured human RPE (hRPE) showed polarity, phagocytic activity, and native RPE characteristic morphology and they were maintained alive for at least 11 days (Warnke et al., 2013). Calejo et al. (2017) developed microporous films comprised of copolymer 96/4 L-lactide/D-lactide with deposited layers of collagen type I and IV. These films simulated BrM characteristics and acted as a support for cultured human embryonic stem cell-derived RPE (hESCs-RPE) cells. These cells were maintained alive for 8 weeks and revealed good adhesion, morphology, and expressed RPE markers. In a different study, fetal human RPE (fhRPE) cultured on porous PCL scaffolds for 8 weeks showed positive results regarding survival, mature and functional RPE markers expression, barrier function, etc. (McHugh et al., 2014). Plasma-modified PDMS with laminin (PDMS-PmL) scaffolds were also used as an artificial BrM. Pluripotent differentiated RPE (dRPE) cells cultured on these scaffolds revealed good adhesion, proliferation, polarization, maturation, and functionality. Also, PDMS-PmL could sustain a multilayer of dRPE cells and precursors of photoreceptors. Furthermore, the PDMS-PmL-RPE subretinal implant *in vivo* in porcine eyes confirmed the biocompatibility of the material for a 2-year period (Peng et al., 2016). In another study, films made of elastin-like recombinamers (ELRs) were investigated: ARPE-19 cells cultured with a bioactive sequence (RGD), maintained their phenotype for up to 5 days and no cytotoxicity was detected (Srivastava et al., 2011).

Stanzel et al. (2014) tested the viability of a subretinal transplant of hESCs-derived RPE cells on a polyester matrix in rabbits. 1-month post-implantation, hESCs-derived RPE remained viable, maintaining their polarization and monolayer structure. hESCs-derived RPE cultured on ultrafine porous polyimide (PI) membranes survived for 6 weeks after subretinal injection in rats, which also showed some functional rescue of electroretinography signal (Ilmarinen et al., 2015). hESCs-RPE on PI transplanted subretinally into rabbit’s eyes showed good membrane tolerability but low RPE cells survival (Ilmarinen et al., 2015).

Spider silk, a very promising biomaterial for artificial BrM, was also tested *in vitro*. This silk fibroin-based membrane allowed ARPE-19 cells to develop a morphology and physiology very similar to those of native RPE cells, as it was shown by the morphological studies and the analysis of their protein expression profiles. This native-like phenotype remained in the silk fibroin films for up to 7 days (Harris et al., 2019). In a very recent study (Jemni Damer et al., 2020) built a multilayer 3D biohybrid retina with RPE, Müller and retinal neural cells on silkworm silk fibroin biofilms, glued with silk fibroin hydrogels. Both, RPE and neural retinal cells survived *in vitro* for the 7 days of the experiment.

In addition to the above substrates, expanded PTFE (ePTFE)-modified membranes were developed to serve as a substrate for RPE growth (Krishna et al., 2011). The surface of the membranes was modified by an ammonia gas plasma treatment, which resulted in ARPE-19 cells attachment and enhanced proliferation. ARPE-19 cells grew in a monolayer and displayed phagocytic capacity. Parylene polymer has also been used as support for the growth and subretinal implantation of hESCs-RPE cells in rats, allowing post-implant cell survival (Hu et al., 2012).

Hsiung et al. (2015) showed that RPE cells derived from hESCs, implanted in the subretinal space within a supporting biomaterial, had higher survival rates than injections of cell suspensions. Previous studies testing artificial RPE strata built with hESCs growing on thin polymeric sheets obtained good results (Redenti et al., 2008; Liu et al., 2012, 2013). Functional integration into the host system is not easy, as it requires migration to the target nuclei, differentiation into the correct cell type for integration into the existing circuitry, and restoration of long-term function. All these steps have to be done by the stem cells while exposed to the hostile conditions of the degenerating retina. Almost all current tissue reconstruction approaches are in the experimental phase, either *in vitro* or *in vivo*, with only a few of these are in clinical trials (see Tables 3, 4).

**TABLE 3:** Biohybrids/TI, Biomaterials-encapsulated RPE cells, *in vitro* studies.

Product	Cell types	Description	Format	Cell survival
Collagen-PLGA [1]	Human RPE cells	RPE cells formed a monolayer, showed polarity and native-like morphology could phagocytose	film	<2 w
Collagen [2]	Human RPE cell line (ARPE-19)	RPE cells formed a monolayer with appropriate phenotype and could phagocytose photoreceptors outer segments	scaffold	>1 w
Collagen [3]	Human RPE cell line (ARPE-19)	RPE cells formed a monolayer on both materials, collagen demonstrated upregulation of angiogenic molecule	scaffold	0.5 w
Gelatin [4]	Human RPE cell line (ARPE-19)	RPE cells formed a monolayer with carbodiimide cross-linked gelatin membrane	film	0.5 w
Elastin-like recombinamers (ELRs) [5]	Human RPE cell line (ARPE-19)	ELRs were not toxic, ARPE-19 proliferated well and maintained their phenotype	film	<1w
Microphotodiode array (SiO2, Si3N4, Pt, MPDA-Pt) [6]	Porcine RPE cells	RPE cells formed a monolayer with appropriate phenotype, biocompatible and non-toxic	film	<2 w
PLC [7]	Fetal human RPE	RPE cells on nanopatterned porous PLC showed better pigmentation, increased cell density, superior barrier function, up-regulation of RPE-specific genes, etc., than on porous PCL, non-porous PCL, or Costar porous polyester transwells	scaffold	>8 w
PDMS-PmL [8]	Pluripotent cell differentiated RPE cells	dRPE revealed good adhesion, proliferation, polarization, maturation and functionality cultured on PDMS-PmL	scaffold	>3 w
PEGDMA [9]	Adult human RPE; porcine RPE	Over 90% viability; confluent cells expressed F-actin and tight junction	film	1 w
PET/PLGA-PGA NP [10]	Adult human RPE stem cell (hRPESC)	RPE cells formed a monolayer, the scaffold and NP showed no cytotoxicity	scaffold	12 w
PLLA/PLGA [11]	Fetal human RPE	Good properties, cell attachment, and proliferation	film	1 w
PLLA/PLGA [12]	Human primary RPE cells/Porcine RPE cells	RPE cells formed a monolayer, good properties, cell attachment and proliferation	film	2 w
PLDLA/Collagen [13]	Human embryonic stem cell derived RPE cells	Supported cell growth, hESCs-RPE showed good adhesion, morphology and maintained phagocytic capacity	film	8 w
PLGA/PEG/PLA [14]	Human RPE cell line (D407)	Micropatterned synthetic biodegradable polymer film that control RPE cell morphology, allows cell-cell interactions and higher cell adhesion	film	1 w
Polyimide (PI) [15]	Adult human RPE stem cell (hRPESC)	Cells established hexagonal, cobblestone morphology with strong pigmentation, expressed RPE specific markers, and phagocytosed photoreceptor outer segments	scaffold	–
Polytetrafluoroethylene-modified surface [16]	Human RPE cell line (ARPE-19)	ARPE-19 cells grew in a monolayer, showed phagocytic capacity. The film was not toxic.	film	2 w
Silk Fibroin/PLC/Gelatin [17]	Human primary RPE cells	Higher cell growth rate and higher expression of characteristic RPE genes compared to PCL and PCL-silk scaffolds	scaffold	>12 w
Silk fibroin [17] [18]	Human RPE cell line (ARPE-19)	RPE cells formed a monolayer, the material showed biocompatibility and no toxicity	film	>16 w
Spider silk proteins [19]	Human RPE cell line (ARPE-19)	RPE cells formed a monolayer with appropriate phenotype and began to exhibit barrier function properties	film	1 w

*ESC, embryonic stem cells; dRPE, pluripotent differentiated RPE; RPE, retinal pigment epithelium; hESCs-RPE, human embryonic stem cell-derived RPE cells; ARPE-19, human retinal pigment epithelial cell line-19; PCL, poly caprolactone; PET, poly(ethylene terephthalate); PLGA, poly(lactic acid-co-glycolic acid); PGA, poly(glycolic acid); NP, nanoparticles; PLDLA, copolymer 96/4 L-lactide/D-lactide; PLLA, poly(L-lactic acid); PLA, poly(DL-lactic acid); PEGDMA polyethylene glycol dimethacrylate; PDMS-PmL, plasma modified polydimethylsiloxane coated with laminin. [1] (Warnke et al., 2013); [2] (Lu et al., 2007); [3] (Imai et al., 2007); [4] (Lai, 2013): [5] (Redenti et al., 2009); [6] (Guenther et al., 1999; Wu et al., 2007); [7] (McHugh et al., 2014); [8] (Peng et al., 2016); [9] (Singh et al., 2001); [10] (McCormick et al., 2020); [11] (Giordano et al., 1997); [12] (Hadlock et al., 1999); [13] (Calejo et al., 2017); [14] (Lu et al., 2001); [15] (Subrizi et al., 2012); [16] (Krishna et al., 2011); [17] (Xiang et al., 2014); [18] (Chirila et al., 2015); [19] (Harris et al., 2019).*

**TABLE 4:** Biohybrids/TI, Biomaterials-encapsulated RPE cells, *in vivo* studies.

Product	Cell types	Description	Format	Model	Cell survival
Collagen [1]	Human RPE cells	Non-crosslinked collagen + supported RPE integrated with host RPE over crosslinked collagen	scaffold	rabbit	<6 w
Fibrinogen [2]	Human fetal RPE	RPE cells with crosslinked fibrinogen particles survived after transplantation into the subretinal space, retinal degeneration was noted in areas of particles	particles	rabbit	4 w
Gelatin [3]	RPE grafts	Subretinal transplant of an allogeneic RPE grafts embedded in gelatin, no infiltration of the graft site with inflammatory cells	film	pig	12 w
Parylene [4]	hESCs-derived RPE	hESCs-derived RPE survived post-implantation	film	rat	–
Parylene-C [5]	hESC-derived RPE cells	Safe and useful implantation of synthetic sheets seeded with organized retinal cells	film	rat	>1 w
PDMS-PmL [6]	iPSC derived RPE	Subretinal scaffold showed biocompatibility and preserved macular function up to 2 years after implantation with no inflammation	scaffold	pig	*
PET or P(LA-co-CL) [7]	hESC-derived RPE cells	Showed subretinal biocompatibility, some migration of native RPE cells into nanofibers, reactive gliosis with some photoreceptor degeneration	film	rabbit	>2 w
Polyester [8]	hESCs-derived RPE	hESCs-derived RPE maintained their structure and polarity on polyester matrix	matrix	rabbit	4 w
Polyester (PET) [7] [9]	Adult human RPE stem cell (hRPESC) and fetal human RPE stem cells	Subretinal implantation of a polarized monolayer of adult hRPESC-derived RPE, after implantation cells survived and maintained key properties, no graft proliferation.	film	rabbit	>4 w
Polyimide (PI) [10]	hESCs-derived RPE	Membrane well tolerated but hESCs-derived RPE showed loss of pigmentation over time	scaffold	rabbit	–
Silk Fibroin/PLC/Gelatin [11]	Human primary RPE cells	Subscleral implantation with no inflammation or rejection	scaffold	rabbit	>12 w

ESC, embryonic stem cells; RPE, retinal pigment epithelium; hESCs-RPE, human embryonic stem cell-derived RPE cells; iPSC, induced pluripotent stem cell; PDMS-PmL, plasma modified polydimethylsiloxane coated with laminin; PET, poly(ethylene terephthalate); P(LA-co-CL), poly(l-lactide-co-ε-caprolactone); PCL, poly caprolactone. [1] (Bhatt et al., 1994); [2] (Oganesian et al., 1999); [3] (Del Priore et al., 2004): [4] (Hu et al., 2012); [5] (Liu et al., 2012; Lu et al., 2012; Diniz et al., 2013); [6] (Peng et al., 2016); [7] (Liu et al., 2014); [8] (Giordano et al., 1997); [9] (Stanzel et al., 2014); [10] (Ilmarinen et al., 2015) [11] (Xiang et al., 2014). *no cell viability assay performed after implantation.

##### Clinical trials

Clinical trials of RPE transplantation include both, injections of suspensions of RPE cells to the posterior segment of the eye and implants of RPE monolayers (alone or in a substrate) between the retina and the native RPE cells.

Safety and tolerability of subretinally transplanted hESC-RPE cells in suspension in patients with advanced D-AMD has been assessed in a Phase I/II trial (U.S. National Library of Medicine, 2020m). The transplanted cells were well tolerated up to 4 months post-injection (Schwartz et al., 2012) and no adverse effects were detected in the patients in the next 22 months (Schwartz et al., 2015). Another Phase I/II Trial with the same objective is currently recruiting patients (U.S. National Library of Medicine, 2020k).

Regarding RPE monolayers transplantation, a Phase I Trial tested the safety of an ESCs-RPE patch on a vitronectin-coated polyester membrane subretinally implanted in two patients affected by W-AMD (U.S. National Library of Medicine, 2020c). Implants were well tolerated and resulted in a gain in visual acuity in both patients (da Cruz et al., 2018). A Phase I/II Trial to assess the safety and tolerability of subretinally implanted hESCs-RPE on a non-degradable Parylene membrane in D-AMD patients is underway (U.S. National Library of Medicine, 2020m).

Parylene and PLGA membranes are in Phases I/II and Phase I/II Clinical Trials, respectively (Liu et al., 2012; Lu et al., 2012; Diniz et al., 2013; U.S. National Library of Medicine, 2020m). A Phase I/II Trial to assess the safety and tolerability of subretinally implanted hESCs-RPE on a non-degradable Parylene membrane in D-AMD patients is underway (U.S. National Library of Medicine, 2020m). Also, Phase I/II Trial for the subretinal implant of a PLGA biodegradable scaffold, carrying autologous iPSC-derived RPEs in patients affected by AMD-associated geographic atrophy is currently recruiting patients (U.S. National Library of Medicine, 2020d).

It is worthy to state that a sheet of iPSC-derived RPE implanted in a W-AMD patient after removal of the neovascular membrane did not show visual acuity improvement and provoked a macular edema 1 year after surgery (Mandai et al., 2017a), while a Japanese Phase I Trial of RPE monolayers of iPSC-derived RPE cells, implanted as strips with no substrate, began in early 2014 was suspended in that same year.

Resuming, up to now, both, RPE cells injected in suspension and implanted as monolayers on artificial BrMs showed good preliminary results in terms of safety and tolerability. RPE cells injected in suspension have several drawbacks such as the necessity to migrate from the injection site and the tendency of suspended RPE to de-differentiate, problems not present in implanted scaffolded RPE monolayers. No positive results are available in terms of AMD therapy and visual acuity recovery.

#### Photoreceptors’ Transplantation

##### General considerations

Different types of cells have been used for neural retinal cell replacement, including photoreceptor precursor cells (PPCs), photoreceptors, and retinal stem cells (RSCs) (see (Lakowski et al., 2011; Tucker et al., 2011; Eberle et al., 2012, 2014; Pearson et al., 2012; Barnea-Cramer et al., 2016) for the former, (Yang et al., 2010; Gust and Reh, 2011; Pearson et al., 2012; Singh et al., 2013; Gagliardi et al., 2018; Garita-Hernandez et al., 2019; Lorach et al., 2019) for the second and (Singh et al., 2020) for the latter. The sources of RSCs can be either endogenous, including neuronal stem cells, or exogenous, including ESCs and iPSCs (Tu et al., 2019).

Similarly to other types of cells, also photoreceptors can be transplanted, either to secret immunomodulatory and/or neuroprotective factors that foster the protection and recovery of the damaged cells or to replace the damaged photoreceptors by new ones (Singh et al., 2020). Photoreceptors’ replacement can be a good therapeutic strategy when, in advanced AMD stages, this type of cells become dysfunctional and eventually die as a result of RPE cells’ deterioration and death (Bhutto and Lutty, 2012). Fortunately, there is a wide time-window for therapeutic interventions, because inner retinal layers’ neurons can survive for prolonged periods of time after photoreceptors degeneration, offering the possibility for cell transplantation to be successful (Medeiros and Curcio, 2001; Jones et al., 2003). However, to make synapses with the bipolar cells and to reverse the synaptic remodeling that takes place after photoreceptors degeneration, implanted photoreceptors need to be functional, a very hard to achieve condition (Jones et al., 2012; Mandai et al., 2017a).

Although post-mitotic transplanted photoreceptors have been proved to be able to regain relative vision functionality (Pearson et al., 2012; Barber et al., 2013), to date no one has achieved a significant restoration of visual function.

##### *In vitro* and pre-clinical trials

###### Photoreceptor transplantation without biomaterials

Similarly to RPE cells, photoreceptors transplantation to the diseased eye can be done either by injection of dissociated cells suspension (see Table 5) (Gust and Reh, 2011; Pearson et al., 2012; Barber et al., 2013; Gagliardi et al., 2018; Garita-Hernandez et al., 2019) or by implanting them as a sheet of tissue, cultivated on a biomaterial substrate (Yang et al., 2010). If the outer nuclear layer, the layer of the retina formed by the bodies of rods and cones, has already degenerated, the latter strategy would be preferable due to the difficulty of the injected cells to get integrated to the damaged tissue and reconstruct a new layer.

**TABLE 5:** Transplanted photoreceptors.

Source	Cell type	Description	Format	Model	Cell survival
E12.5-P28 days mice [1]	photoreceptors	Cells integrated well in the host retina and expressed equal markers	dissociated cells	mouse	2 w
hiPSCs [2]	photoreceptors	Photoreceptors survived and matured in the host eye	injected in clusters	rat	10 w
P4 mice/hiPSCs [3]	photoreceptors	Blind mice recovered visual function/mice showed signs of retinal repair	cell suspension	mouse	4 w
P4-8 mice [4]	Rod precursors	Rod precursors stablished synaptic connection with cells in retina of Gnat12/2 mice and were light-responsive	dissociated cells	mouse	6 w
P6-8 mice [5]	photoreceptors	Prph2^+/Δ307^ model showed higher integration of rods	cell suspension	mouse	4 w
P8 rat [6]	photoreceptors	Transplantation reduced cone loss 6 months after surgery	sheet	rat	24 w
Adult rats [7]	Full-thickness retina with attached RPE	well-preserved host photoreceptor layer and RPE integrated well	graft	rat	–

*E, embryonic day; P, postnatal day; Gnat12/2 mice, a model of congenital stationary night blindness; hiPSCs, human induced pluripotent stem cells; Prph2^+/Δ307^, model of retinitis pigmentosa. [1] (Gust and Reh, 2011); [2] (Gagliardi et al., 2018); [3] (Garita-Hernandez et al., 2019); [4] (Pearson et al., 2012); [5] (Barber et al., 2013); [6] (Yang et al., 2010); [7] (Lorach et al., 2019).*

Subretinal injection of a suspension of photoreceptors from embryonic-postnatal mice showed that donor cells integrated well in the host retina and the morphology and marker expression of donor cells were similar to those of the photoreceptors of the host retina (Gust and Reh, 2011).

Early postnatal rat photoreceptors implanted as a sheet to a model of dominant retinitis pigmentosa slowed cone loss 6 months after surgery (Yang et al., 2010). Rod precursors from postnatal mice injected as dissociated cells formed synaptic connections with bipolar and horizontal cells in the retina of Gnat12/2 mice (congenital stationary night blindness model) and responded to light stimuli (Pearson et al., 2012). Also, early postnatal mice rod-photoreceptors, transplanted as a cell suspension into 6 different mice models of retinal degeneration, got integrated into the host tissue with better results in the Prph2^+/Δ307^ model (retinitis pigmentosa model) (Barber et al., 2013). hiPSC-derived CD73+ photoreceptors isolated and injected in clusters in the eyes of immunosuppressed rats survived, and matured for 10 weeks (Gagliardi et al., 2018). Rat RPE/photoreceptors’ grafts transplanted in a retinal degeneration rat model protected the host photoreceptor layer from degeneration 6 months after surgery. The graft integrated well with the host tissue and created synapses with the bipolar cells (Lorach et al., 2019).

An interesting approach was taken by Garita-Hernandez et al. (2019). They introduced a hyperpolarizing microbial opsin in photoreceptors extracted from P4 mice and transplant them as a cell suspension into transgenic blind mice lacking the whole layer of photoreceptors (Cpfl1/Rho-/- and C3H rd/rd mice) who recovered their visual function. The same authors obtained hiPSCs-derived cones expressing NpHR (Natronomonas pharaonis halorhodopsin); blind mice that received them also as cell suspension, displayed signs of retinal repair 4 weeks post-injection (Garita-Hernandez et al., 2019).

###### Photoreceptor transplantation with biomaterials

Both synthetic polymers and biological materials can be used in the fabrication of scaffolding structures for photoreceptors implants: natural materials like alginate, collagen, etc., are suitable for imitating natural architectures (Ramakrishna et al., 2001); synthetic polymers, such as poly-caprolactone (PCL) and poly (lactic-co-glycolic) acid (PLGA), offer higher mechanical strength and controllable degradation rates (Chen et al., 2011) although the latter usually cause less biological activity (Yang et al., 2017).

Scaffolds for retinal progenitor cells (RPCs) transplants can be cylindrical, mimicking the vertical disposition of cells in the retina, fibrous, mimicking the microstructure of the extracellular matrix or made by hydrogels, to mimic the mechanical properties of the retina (Kador and Goldberg, 2012). The employment of 2D–3D scaffolds has been proved to be an efficient strategy to overcome the limitations of free cell injections, like low survival, low cell integration at the injection site and difficulty to maintain the injected cells in the target areas (Behtaj et al., 2020).

A 3D biodegradable poly-caprolactone (PCL)-microfabricated scaffold promoted good RPCs retention *in vitro* (Sodha et al., 2011). Another biodegradable PCL scaffold, in this case subretinally implanted in porcine eyes, showed good tolerability without retinal neither choroid inflammation (Christiansen et al., 2012). PCL scaffolds with varying surface topographies enhanced mice RPCs differentiation toward photoreceptor phenotype *in vitro*. Transplanted RPCs-PCL integrated well into the host retina and expressed specific markers of photoreceptors (Yao et al., 2015). And (Lawley et al., 2015) found that vitronectin-simulating peptides into a PCL film increased hRPCs adhesion, inhibited hRPCs proliferation and induced differentiation to photoreceptors phenotypes. Transplanted cells into mice degenerating retina, migrated to the outer nuclear layer and survive for 3 weeks.

In a multiple substrates study, RPCs showed good adhesion on poly-l-lysine, fibronectin, laminin, hyaluronic acid, and matrigel substrates *in vitro* (Thakur et al., 2018).

The fabrication of hybrids has also been explored. Poly-caprolactone (PCL)-retinal extracellular matrix hybrids allowed adhesion of the hRPCs cells and helped their differentiation toward the photoreceptor phenotype *in vitro* (Baranov et al., 2014). Interphotoreceptor matrix employed as a scaffolding material for hRPCs showed good cellular attachment absence of cytotoxicity and hRPCs differentiation toward the photoreceptor phenotype *in vitro* (Kundu et al., 2018). Tucker et al. (2010) tested a biodegradable cell delivery platform made of pre-activated MMP2 into a poly(lactic-co-glycolic) acid polymer *in vitro* and *in vivo*. Active MMP2 was released in a controlled manner and allowed RPCs to migrate into the outer nuclear layer and adopt a photoreceptor phenotype.

Jung et al. (2018) developed a 3D cup-shaped scaffold with μm-sized structures in ultrathin biocompatible elastomer films. These films were composed of a non-biodegradable part plasma-modified polydimethylsiloxane (PDMS) and a biodegradable part poly (glycerol-sebacate) (PGS) and were designed to deliver photoreceptors in a polarized manner. hPSCs-PRs showed polarization and robust survival *in vitro* 3 months post-seed in the scaffold.

Table 6 shows the most relevant studies for biomaterials-encapsulated progenitor cells/photoreceptors.

**TABLE 6:** Biohybrids/TI.

Product	Cell types	Description	Format	Model	Cell survival
Hyaluronic acid/methylcellulose [1]	RSC-derived rods	Improved cell survival, integration and migration, improved rod survival and visual function	hydrogel	mouse	3 w
Hyaluronic acid/methylcellulose [2]	RSCs	Superior cell distribution in subretinal space	hydrogel	mouse	4 w
IPM [3]	hRPCs	hRPCs attached well and differentiated into photoreceptors IPM showed no cytotoxicity	scaffold	*in vitro*	1 w
MMP2–PLGA polymer [4]	RPCs	RPCs differentiated into photoreceptors	scaffold	*in vitro*	2 w
MMP2–PLGA polymer [4]	RPCs	RPCs differentiated into photoreceptors and migrated to the outer nuclear layer of mice retina	scaffold	mouse	2 w
PCL [5]	Primary mouse embryonic RPCs	Localized to the outer nuclear layer and expressed appropriate photoreceptor markers	film	mouse	–
PCL [6]	Primary mouse embryonic RPCs	Supported cell growth, some migration and differentiation	scaffold	mouse	>4 w
PCL-extracellular matrix of the retina [7]	hRPCs	hRPCs adhered well and differentiated to photoreceptors	scaffold	*in vitro*	1 w
PLLA/PLGA [8]	GFP+ mouse RPCs	RPCs-seeded demonstrated effectiveness and increasing progenitor cell survival	scaffold	mouse	4 w
Polycaprolactone [9]	RPCs	Scaffold showed good retention of cells and good permeability	scaffold	*in vitro*	1 w
Polycaprolactone [10]	RPCs	RPCs integrated well in the outer nuclear layer and showed photoreceptor fate markers	scaffold	mouse	3 w
Polydimethylsiloxane and poly(glycerol-sebacate) [11]	hPSCs-PRs	Good polarization of PRs and robust survival 3 months post-seed	scaffold	*in vitro*	12 w
Poly (glycerol sebacate) [12]	Primary mouse embryonic RSPC	Transplanted cell migration into retina and maturation	scaffold	mouse	4 w
Poly-L-lysine, fibronectin, laminin, hyaluronic acid, and matrigel [13]	RPCs	RPCs showed good adhesion in the named substrates	scaffold/matrix	*in vitro*	–
Poly (methyl methacrylate) (PMMA) [14]	GFP+ mouse RSPC	PMMA scaffolds and transplanted into the sub-retinal space, biocompatible and non-toxic, retained RPE cells better during transplant, integrated cells expressed mature and immature markers	scaffold	mouse	1 w
Vitronectin- PCL [15]	hRPCs	hRPCs differentiated into photoreceptors and migrated to the outer nuclear layer of mice retina	film/scaffold	mouse	3 w

*Biomaterials-encapsulated progenitor cells/photoreceptors. RPE, retinal pigment epithelium; RSCs, retinal stem cells; hRPCs, human retinal progenitor cells; RPCs, retinal progenitor cells; IPM, interphotoreceptor matrix; MMP2, matrix metallopeptidase 2; PLGA, poly(lactic acid-co-glycolic acid); PCL, poly caprolactone; PLLA, poly(L-lactic acid); PMMA, poly(methyl methacrylate); GFP, green fluorescent protein; hPSCs-PRs, human progenitor stem cells-derived photoreceptors. [1] (Shoichet et al., 2015); [2] (Ballios et al., 2010); [3] (Kundu et al., 2018): [4] (Tucker et al., 2010); [5] (Yao et al., 2015); [6] (Redenti et al., 2008); [7] (Baranov et al., 2014); [8] (Tomita et al., 2005); [9] (Sodha et al., 2011); [10] (Yao et al., 2015); [11] (Liu et al., 2014; Stanzel et al., 2014); [12] (Redenti et al., 2009); [13] (Thakur et al., 2018); [14] (Tao et al., 2007); [15](Lawley et al., 2015).*

##### Clinical trials

Although preclinical studies have shown that all, RPCs, photoreceptor precursors and mature photoreceptors could potentially replace damaged retinal cells, clinical trials for these approaches are limited.

A Phase II study in which human fetal neural retinal tissue and RPE were transplanted together in a group of AMD patients, showed promising results (Radtke et al., 2008; U.S. National Library of Medicine, 2020o). Interestingly, the vision improvement observed in treated patients began 6 months after transplantation, the time predicted for the fetal cells to differentiate into mature photoreceptors. Another phase I/II study used HuCNS-SC^®^ (human central nervous system stem cells) injected subretinally in AMD patients (U.S. National Library of Medicine, 2020r). This study was completed in 2015 but no results have been posted yet. Besides, a long-term follow-up safety study from the same sponsor has terminated based on, as posted, a business decision unrelated to safety concerns (U.S. National Library of Medicine, 2020i). Another clinical trial using HuCNS-SC^®^ was terminated due to the same reason (U.S. National Library of Medicine, 2020q).

Bone marrow-derived stem cells have also got promising results in a Phase I/II Trial: NCT01920867 (Weiss et al., 2015; U.S. National Library of Medicine, 2020p). Another Phase I/II Trial testing bone marrow-derived stem cells, proved their safety: NCT01518127 (Cotrim et al., 2017; U.S. National Library of Medicine, 2020h).

Three ongoing clinical trials are using fetal-derived RPC for photoreceptor’s replacement in retinitis pigmentosa patients. ReNeuron is carrying a phase I/II trial to evaluate the safety, tolerability and preliminary efficacy of hRPCs, administered as a suspension by a single subretinal injection (U.S. National Library of Medicine, 2020l). An already completed phase I/II study testing hRPCs injected intravitreally, showed that hRPCs were safe and well-tolerated at doses up to 3 million cells (U.S. National Library of Medicine, 2020n). A phase IIb clinical trial designed to assess efficacy of hRPCs is currently active (U.S. National Library of Medicine, 2020j). A phase I study to determine the safety of CD34+ marrow-derived stem cells injected intravitreally into the eye as a treatment for patients with various retinal conditions started in 2012 (U.S. National Library of Medicine, 2020f). Not results have been posted regarding the enrolled AMD patients. Future results from these studies may be extrapolated to AMD patients.

#### Retinal Organoids and 3D Structures Transplantation

Retinal cells’ transplantation low success rates are due to the above mentioned adverse retinal environmental conditions, but also to the complexity of the interactions between photoreceptors and other retinal cell types as well as to the structural complexity of the photoreceptors themselves (Llonch et al., 2018). Particularly critical are the interactions RPE-photoreceptors since RPE cells are the responsible for the recycling of the visual pigment, for the phagocytosis of photoreceptors’ external segment as well as for the secretion of growth factors (Strauss, 2005). Indeed, co-culture studies, attempting at de-differentiating mesenchymal stem cells, showed that RPE cells determine both, cell differentiation and connectivity of the differentiated cells (Salero et al., 2012). Unfortunately, during culture passages, RPE cells easily undergo an epithelial-mesenchymal transition (EMT), losing their characteristic cell polarity and cell-cell adhesion, necessary for the survival and functional integration of the photoreceptors (Sonoi et al., 2017). Furthermore, RPE cell cultures should be prepared exclusively with macular RPE cells because of phenotypes’ differences between cells located in the peripheral part and cells located in the posterior part -macula- of the eye (cell shape and size, proliferation capacity, maturation time, granule content, growth potential, timing for the formation of tight junctions, etc. (Burke and Hjelmeland, 2005; Haderspeck et al., 2019). Non-homogeneous cultures would lead to retinal-type tissues, with structure vulnerable to degenerative retinal diseases (Burke and Hjelmeland, 2005).

Due to the above-mentioned complications and to the biological limitations of the available cells, researchers started to build artificial retinal tissues, with structural complexity and functionality mimicking the natural ones. As a consequence, retinal organoids have been built from either ESCs or iPSCs, by differentiating retinal cells through complex experimental protocols in 3D cell cultures (Eiraku et al., 2011; Nakano et al., 2012; Llonch et al., 2018).

The main benefits of 3D cultures are a high diversity of cell types, better tissue survival, functional interaction between the different types of cells, and the achievement of retinal layers thanks to the self-organizing capabilities of the PSCs. In addition to their use for therapeutic purposes, 3D cultures represent a model of great interest for the study of retinogenesis, a non-animal model of retinal diseases and a perfect tissue for drug screening. The main limitations of these types of models are the lack of vascularization, which leads to long-term necrosis in the innermost areas of the organoid, the lack of interaction between the RPE and photoreceptors’ layer, the maturation of the different cell types which does not reach an adult state and the absence of some types of important cells, like microglia (Achberger et al., 2019). Furthermore, all organoids have a characteristic folded structure that not only makes implantation difficult but also causes greater cell death in the internal layers of this artificial tissue. The use of biomaterials could help in developing flat vascularized organoid retinas that would favor the interaction with the host tissue as well as the acceptance and survival of the transplanted cells (Singh D. et al., 2018). Advanced biomaterials could also facilitate artificial tissues manipulation and implant in a specific place in the host retina (Singh R. et al., 2018).

Tables 7, 8 resume up-to-day research in retinal organoids.

**TABLE 7:** *In vitro* studies of 3D structures/organoids.

References	Product	Cell type	Description	Cell survival
Eiraku and Sasai, 2012	Matrigel	mESC	Organoid model to obtain a 3D retinal model.	>3 w
Hiler et al., 2015	–	miPSC	Model obtained from iPSC derived from different sources to study retinogenesis.	4 w
Hasegawa et al., 2016	Collagen	mESC	Organoid gel model to study retinogenesis.	>1 w
Völkner et al., 2016	Matrigel	mESC	Model to study retinogenesis.	3 w
Chen et al., 2016	–	mESC miPSC	Model to study morphogenesis of the photoreceptors.	5 w
Lamba et al., 2006	Matrigel	hESC	Model to obtain neural precursors and co-culture with retinal explant.	3 w
Osakada et al., 2008	–	mESC monkey ESC hESC	Model to obtain retinal cells. Co-culture with retinal explants to study interaction.	mESC-PR: >4 w monkey ESC-RPE:13 w monkey ESC- PR: 18 w hESC- RPE: 17 w hESC – PR: 28 w
Ohlemacher et al., 2015	Matrigel	hPSC	Model to study development of retinal tissue and drug screening.	10 w
Fligor et al., 2018	Matrigel	hPSC	Model to study development of retinal tissue and retinal ganglion cells growth.	6 w
Zhong et al., 2014	Matrigel	hiPSC	Modeling of diseases and to obtain retinal cells.	13 w
Nakano et al., 2012	–	hESC	Model to obtain and store retinal cells.	18 w
Lowe et al., 2016	Matrigel	hESC	Organoid model.	2 w
Achberger et al., 2019	Agarose Polydimethylsiloxane and Polyethylene terephthalate	iPSC	Development of vascularization in an organoid matrix and membrane models.	2 w
Thangaraj et al., 2011	–	Chicken retina	Organotypic culture. Retinal development model.	3 w
Engelsberg et al., 2008	Millicell inserts	Human retina	Organotypic culture. Development of neuronal and glial cells.	6 w
Niyadurupola et al., 2011	Millicell inserts	Human retina	Organotypic culture. Model for retinal ganglion cell degeneration.	0.5 w
Osborne et al., 2016	–	Human retina	Organotypic culture. Model for retinal ganglion cell degeneration.	4 w
Curatola et al., 2005	–	Mice retina	Organotypic culture. Vascularization of the 3d culture.	2 w
Caffé et al., 2002	–	Mouse retina	Organotypic culture. Retinal development.	4 w
Ghosh et al., 2009	–	Rat retina	Organotypic culture. Isolation of photoreceptors.	2 w
Wang et al., 2002	–	Mice retina	Organotypic culture. Introduction of genes. Study cellular differentiation.	1 w
Subrizi et al., 2012	Polyimide	hESC	Development of retinal pigment epithelium in a polyimide membrane. Coculture with rat retinal explants.	hESC-RPE: 2–8 w Coculture: <0.5 w
Johnson et al., 2016	–	Mice retina	Organotypic culture. Neuroprotection studies.	1 w
Engelsberg et al., 2005	–	Porcine retina	Organotypic culture.	6 w
Taylor et al., 2013	–	Porcine retina	Organotypic culture. Study the effect of the neurotrophic factors in retinal survival.	1.5 w
Taylor et al., 2014	polycarbonate	Porcine retina	Organotypic culture in a polycarbonate membrane. Study the effect of the biomaterial in the structure of the retina.	1.5 w
Huang et al., 2000	–	Rabbit retina	Organotypic culture. Developmental study.	1 w
Koizumi et al., 2007	–	Rabbit retina	Organotypic culture. Model for genetic manipulation.	1 w

*mESC, mouse embryonic stem cell; miPSC, mouse induced pluripotent stem cell; hESC, human embryonic stem cell; hiPSC, human induced pluripotent stem cell.*

**TABLE 8:** textitIn vivo studies of 3D structures/organoids transplantation.

References	Product	Cell type	Description	Animal	Cell survival
Gonzalez-Cordero et al., 2013	–	mESC	Transplantation of photoreceptors from an organoid model.	Mouse	3 w
Assawachananont et al., 2014	Matrigel	mESC miPSC	Transplantation of retinal sheet into a degenerative model.	Mouse	2–24 w
Decembrini et al., 2014	–	mESC	Organoid model for retinal cells transplantation.	Mouse	3 w
Mandai et al., 2017a	–	miPSC	Retinal tissue transplant in a degenerative model.	Mouse	4 w
Johnson and Martin, 2008	–	Rat retina h-müller cells	Organotypic culture. Transplant of cells in an ocular hypertension model.	Rat	>2 w
Santos-Ferreira et al., 2016a	Matrigel	mESC	Organoid model. Transplantation of photoreceptors into mouse models.	Mouse	4–24 w
Santos-Ferreira et al., 2016b	Matrigel	mESC	Organoid model. Transplantation of photoreceptors into mouse models.	Mouse	10 w
Singh D. et al., 2018	Gelatin Chondroitin Sulfate Hyaluronic Acid	hESC	Biomaterials are used to develop a flat sponge retinal organoid that improves implantation and interaction with host tissue. Subretinal implant.	Mouse	12 w
Julien et al., 2011	Polyimide, gelatin	Rat retina	Subretinal implantation of organotypic layers in a biofunctionalized membrane maintaining a laminated format (Foils).	Rat	1 w (*in vitro*) 9 w (*in vivo*)
McLelland et al., 2018	–	hESC	Subretinal implantation or retinal sheets from organoids integrate in the host tissue.	Rat	8–43 w
Singh et al., 2019	–	hESC	Subretinal implantation or retinal tissue.	Cat	4–9 w
Koss et al., 2016	Parylene C	hESC	Subretinal implantation of a layer of RPE on mesh.	Yucatan minipig	4 w
Xian et al., 2019	Poly (Lactic-Co-Glycolic) (PLGA)	hiPSC	Epiretinal transplantation of retinal organoids using PLGA-composites.	Monkey	8 w
Mandai et al., 2017b	–	hiPSC	Transplantation of a retinal pigment epithelial cells sheet in a AMD model.	Human	52 w

*mESC, mouse embryonic stem cell; miPSC, mouse induced pluripotent stem cell; hESC, human embryonic stem cell; hiPSC, human induced pluripotent stem cell.*

## Discussion

### Functional Integration of the Implanted Cells

Cell therapy, and in particular stem cell-based therapy, is gaining strength as a potential therapeutic approach for untreatable retinal degeneration diseases (Bharti, 2018; Singh et al., 2020). It consists either in the use of ocular multipotent or RPCs, administered in a non-polarized way (cell suspension), to provide non-selective neuroprotective and/or neuromodulator factors, or in the implant of layered constructs of cells aiming at replacing the damaged or degenerated retinal tissue. In both cases, therapeutic effects are conditioned by the limited cell survival within the harmful ocular environment (Singh et al., 2020).

In the majority of the preclinical trials with dissociated cells suspensions for photoreceptor regeneration, the transplanted cells fail to survive or to functionally integrate with the host tissue; despite some positive results none of the attempted approaches achieved a significant restoration of the visual function (Gamm and Wong, 2015; Canto-Soler et al., 2016; Zarbin, 2016). Furthermore, it is not known to which extent the observed improvements are due to the capability of the grafted cells of replacing the lost retinal tissue; to the secretion of protective and regenerative factors that protect the retinal neurons, or by both of them (Liu et al., 2020).

Regarding RPE, there are several investigations on the inoculation of suspensions of this type of cells. However, up-to-now evidence suggests that suspension-injected RPEs do not consistently form a monolayer and do not survive in the long term (Hu et al., 2012; Diniz et al., 2013). Since RPE cells’ therapeutic effects are conditioned by the polarization of the implanted cells as well as by their spatial arrangement in a monolayer settled in the subretinal space, there is a great challenge for injections of suspended cells (Miyagishima et al., 2016, 2017). Injected in suspension RPE cells loose most of their morphological and functional properties as proved by the low efficiency of this therapy in both, preclinical and clinical trials (Singh et al., 2020). The most promising studies focus on tissue transplantation or patches of RPE.

Promising results in both, feasibility and functionality, have been obtained in preclinical and clinical trials using functional RPE derived from both iPSCs (Bracha et al., 2017; Mandai et al., 2017b) and ESC cells (Klimanskaya et al., 2004; Bharti et al., 2006; Idelson et al., 2009; Miyagishima et al., 2016; May-Simera et al., 2018), as well as RPESC (Salero et al., 2012; Blenkinsop et al., 2013; Miyagishima et al., 2016; Mandai et al., 2017b). However, the use of iPSCs and ESC cells can lead to abnormal cell growth in the retina. Moreover, the differentiation toward a specific cell type (RPE) may not be complete or efficient, thus hindering their integration in the host retina. On their side, RPESC, although being fully committed to the RPE lineage, they are extracted from cadavers, which can lead to the retention of endophenotypes of aging and disease. Unlike cell suspensions, where the characteristics of RPE are lost, cell patches are capable of maintaining many of the typical cell functions (Miyagishima et al., 2016, 2017) which makes autologous choroid-RPE transplant surgical procedures a good candidate for AMD treatment (Joussen et al., 2006; Maaijwee K. et al., 2007; Maaijwee K.J.M. et al., 2007; Maaijwee et al., 2008).

Recent studies on subretinal injection of a suspension of photoreceptor precursors derived from mouse or human ESC/iPSC in rodent models of photoreceptor degeneration, suggested that the implanted cells are capable of getting integrated into the host retina, of achieving a morphological and functional differentiation similar to the native photoreceptors as well as of restoring some visual abilities (Lamba et al., 2009; Gonzalez-Cordero et al., 2013; Homma et al., 2013; Decembrini et al., 2014; Barnea-Cramer et al., 2016; Santos-Ferreira et al., 2016b). However, only a low proportion of transplanted cells can get integrated into the retina. What really happens is an exchange of intercellular materials between the immense majority of the native photoreceptors and the transplanted cells (Pearson et al., 2016; Santos-Ferreira et al., 2016b; Singh et al., 2016). This material transfer is bidirectional and, interestingly, it is independent of the source of implanted stem cells (Singh et al., 2016; Ortin-Martinez et al., 2017; Waldron et al., 2018). It seems that the therapeutic effects are due more to neurotrophic and neuroprotective function, rather than functional integration in the diseased retina (Liu et al., 2020). These facts raise the need to reevaluate all up-to-day photoreceptor transplant studies, characterizing the relative contribution to vision restoration based on material transfer and donor integration.

The recent development of 3D technology for the culture and differentiation of ESC/iPSC in retinal tissue has allowed several groups to test the viability of transplanting retinal sheets derived from this type of stem cell (Assawachananont et al., 2014; Shirai et al., 2016; Mandai et al., 2017a; Iraha et al., 2018). These ESC/iPSC-derived retinal sheets, derived from both mice and humans, are capable of surviving in the host retina for up to 6 months after transplantation in the subretinal space, in animal models of end-stage photoreceptor degeneration. In addition, they manage to generate all types of neural cells in the retina, i.e., photoreceptors, bipolar, amacrine, and retinal ganglion cells. In particular, the photoreceptors achieved a high degree of maturity, expressing opsins, synaptic proteins and formation of inner and outer segments (Assawachananont et al., 2014; Iraha et al., 2018). However, it has been revealed that in these grafts it is not possible to maintain an adequate laminar organization; instead, they generate a disorganized histoarchitecture (Assawachananont et al., 2014; Shirai et al., 2016; Mandai et al., 2017a; Iraha et al., 2018). 3D-culture techniques and implantation of retinal sheets suppose an expectation of providing photoreceptors in the form of tissue, providing them with an improved physical and physiological microenvironment, increasing their survival and functional integration capabilities. However, this area of cell therapy research has only just begun, so there are many emerging challenges and limitations that need to be addressed.

Regarding retinal organoids, despite the great advances achieved in this field, a number of serious limitations impede the employment of this technology for real AMD therapies: (1) lack of vascularization, which leads to long-term necrosis in the innermost areas of the organoid (2) lack of interaction between RPE and photoreceptor layer, essential for the proper functioning of the neurons (3) impossibility of the different cell types to reach an adult state, despite the complex differentiation protocols that are carried out and (4) lack of some essential cell types, such as microglia, despite presenting a high number of cell types (Achberger et al., 2019).

The lack of certain cell types during the development of the 3D culture may be due, among other causes, to the lack of sufficient physical and environmental signals, also causing high variability between the various retinal organoid models. A possible solution to some of the limitations mentioned above in relation to this type of culture could be the use of biomaterials as a support for the organoid’s growth and differentiation and their biofunctionalization to make them able to generate the adequate physical and chemical signals (molecules of the extracellular matrix) to stimulate the correct differentiation of the cells (Hynes and Lavik, 2010). Biomaterials could also allow interaction between the various layers of the retina, increasing connectivity and synaptogenesis, favoring obtaining a functional and complete retina (Singh R. et al., 2018).

Summarizing, despite the promising results obtained in several studies, long-term viability of cell implants has not yet been achieved, neither significant functional integration in the host retina nor improvement of long-term visual capabilities. Up-to-day photoreceptors integration in the host retina is clearly insufficient and/or inadequate. AMD cellular therapy has three main unanswered questions: (1) RPE plays a critical role in the maintenance and function of photoreceptors but very few studies consider the interdependence between RPE cells and photoreceptors (Bhutto and Lutty, 2012; Handa, 2012; Handa et al., 2017) which could be one of the reasons long-term maintenance of RPE and photoreceptor transplantation has not yet been achieved. In this case, a possible solution would be the joint transplantation of the two sheets of cell, either sequentially or at the same time. (2) The mechanisms involved in the development and integration of photoreceptors in the retinal circuit remain unknown (Gamm and Wong, 2015). It has been observed expression of specific synaptic proteins in the terminal bar of the implanted photoreceptors; it has also been observed expression of postsynaptic proteins of the host bipolar cells in contact with the donor cells; and it has been observed a certain improvement of visual function. However, evaluation of the neural function of these transplants are very few and clearly insufficient (Singh et al., 2013). (3) Detailed mechanisms for material exchange between donor and host cells are almost unknown (Lluis and Cosma, 2010; Sanges et al., 2016). Most of the cells identified as integrated donor photoreceptors in the most recent studies could actually be host photoreceptors with exchanged material (Pearson et al., 2016; Santos-Ferreira et al., 2016b; Singh et al., 2016; Ortin-Martinez et al., 2017; Waldron et al., 2018).

Addressing these problems could allow us to identify those factors that could be used to promote cellular viability, synaptogenesis and integration of the donor cells to the host tissue. Further questions regard the safety of the transplants (depending on their origin), possibility of transferring of the pathology to the implanted healthy cells, etc. It should be noticed that the most promising results have been obtained in tissue transplantation of retinal sheets and RPE patches.

### Maintenance of the Immunoprivileged State of the Eye

Several subfoveal RPE implantation techniques have been employed in AMD patients. Most often, excision at sub-macular level is necessary for transplantation of the monolayer of fetal or adult RPE. This procedure can lead to acute tissue inflammation and a consequent eye damage that can impair the visual route, or even a higher immune reaction if the implant comes from another organism. Therefore, knowledge of the immunosuppression mechanisms is necessary to prevent rejection of RPE layers (Tezel et al., 2007), and it has been postulated that the low success rate of RPE monolayers transplants is due to alterations of the immunoprivileged state of the eye (indeed, rejection rate of retina transplants is higher than of other tissues, such as heart or skin (Streilein, 2003; Niederkorn and Kaplan, 2007; Sandhu et al., 2019)). Clinical studies of RPE transplants in patients undergoing immunosuppressive therapy, suggest that the immune system could destroy the transplanted cells without involving any inflammatory process (Schwartz et al., 2015). On the other hand, a higher survival of photoreceptor transplants has been observed in preclinical studies when some type of immune suppression was used after the surgery (Sandhu et al., 2019). Combined RPE-photoreceptors transplants showed that the use of immunosuppressants could improve the cellular integrity of the graft (Radtke et al., 2002, 2008). However, these results have not yet been replicated. In other preclinical studies, it was observed that only 0.5% of photoreceptors got integrated into the retina (Pearson et al., 2016; Santos-Ferreira et al., 2016a). It is not clear if the effects of photoreceptors transplantation in mice are the result of cell integration and synapse formation, rather than cytoplasmic exchange between donor and host cells. However, we can conclude that the use of immunosuppressants in human retinal transplants may lead to a higher success rate.

### Biomaterials and Improvement of Cell Survival

2D – 3D scaffolding approaches have been shown to be efficient for overcoming limitations like low cell survival, lack of cell integration at the transplant site or keeping the injected cells in the target area (Behtaj et al., 2020). However, it is important to mention that the majority of the biomaterials developed for retinal regeneration has not been tested *in vivo*.

Biodegradable polymer scaffolds for the transplantation of retinal stem cells increase survival cell rate (Schubert et al., 2014). On the other hand, the dimensional conformation or porosity of the scaffold seems to promote the union and subsequent differentiation and orientation of the RPCs (Lee Ventola, 2014). Other studies defend the idea that scaffold topography influences orientation, because of its positive influence on differentiation, morphology, proliferation, migration and adhesion of the cells (Ramakrishna et al., 2001; Kador et al., 2016; Yang et al., 2017).

Scaffolds can actively interact with different cellular components. Furthermore, biomaterials can include biological cues (e.g., cellular ligands) to promote cell adhesion or physical clues (e.g., scaffold topography) to favor cell alignment and morphology. Scaffolds can also serve as supplier or repository for excitatory growth signals to accelerate tissue regeneration (Chan and Leong, 2008). Biomaterials with a sophisticated chemical and morphological structure to allow the survival, proliferation and differentiation of RPE cells and the consequent successful transplantation into the degenerated retina have also been pursued (Chichagova et al., 2018). For the encapsulation of iPSC and hESC, optimal and standardized culture media and good bioengineering processes are needed to achieve the desired expansion and differentiation, processes that are currently under investigation (Bracha et al., 2017; Mandai et al., 2017b; U.S. National Library of Medicine, 2020c).

Biomaterials should be biocompatible, biologically inert and sterilizable, not triggering cytotoxic processes neither inflammatory responses while promoting the proliferation, survival and migration of the implanted cells. Significant challenges are the risk of immune reactions at the implant site as well as the long-term survival and functionality of the transplanted cells.

A very promising biomaterial is silkworm silk fibroin, because it fulfills all the above requirements. The encapsulation of mesenchymal stem cells in silk fibroin hydrogels promotes a substantial release of anti-inflammatory and anti-oxidant molecules with marked neuroregenerative and neuroprotective properties that could be used in AMD treatment (Martín-Martín et al., 2019; Jemni Damer et al., 2020).

Current research of cell and tissue engineering approaches focuses on the preservation of photoreceptors by replacing the damaged retinal pigment epithelium since this technique has been shown to slow the degeneration of photoreceptors and even restore partial visual functions (Aboutaleb Kadkhodaeian et al., 2019; Jin et al., 2019; McGill et al., 2019; Zarbin, 2019). The development of physical substrates for artificial RPE sheets is the main objective for biomaterials engineers. It is worth noting that actual focusing on photoreceptor’s preservation is partly due to the current difficulties to maintain functional photoreceptors alive before they could get transplanted, as well as to integrate the transplanted photoreceptors to the neural retina and functionally replace those already lost. In culture, photoreceptors can be maintained alive for 24–48 h, depending on the age of the donor (Fontaine et al., 1998) and less than 20% survive after transplantation (Sortwell et al., 2000). It is unclear how many of these alive cells are functional photoreceptors. State-of-the-art 3D scaffolds have been used to build a polarized photoreceptor monolayer capable to can get integrated into the neural retina, but also to facilitate the differentiation of progenitors of photoreceptor (Jung et al., 2018).

Good results on biomaterials and RPE cells have been obtained from both, *in vitro* and *in vivo* studies (Warnke et al., 2013; McHugh et al., 2014; Stanzel et al., 2014; Ilmarinen et al., 2015; Peng et al., 2016; Calejo et al., 2017; Jemni Damer et al., 2020) although how long the transplanted cells could survive has still to be determined. The scaffolds on which they are placed may be designed to act either as supporting devices or as artificial BrMs, showing the latter the best viability of RPE cells. In clinical trials, RPE cells have been injected in suspension, as a monolayer and also on biomaterials (Schwartz et al., 2015; da Cruz et al., 2018; U.S. National Library of Medicine, 2020s). Unfortunately, these clinical trials are very few and are in very early stages of development, so their results should be analyzed accordingly.

In conclusion, the use of biomaterials to generate implantable biohybrid tissue may be a potential solution to the main problems of cell transplantation, increasing the survival of implanted cells and improving their integration capacity.

## Conclusion

Cell replacement, either by using cell suspensions or by integrating cell’s sheets on a scaffolding material to promote the survival of the transplanted cells, is a promising treatment for advanced AMD stages. Pioneering studies have provided a proof-of-concept for RPE and photoreceptors transplantation as a plausible therapeutic strategy.

The effective integration of the transplanted cells into the damaged retina is the principal limitation of cell therapy. The development of advanced biomaterials to support the *in vitro* development of artificial 3D retinal tissues, as well as the implantation and the functional integration of these constructs into the damaged retina represents the most promising therapeutic approach. Indeed, there is evidence that retinal cells on supporting biomaterials, implanted in the posterior segment of the eye, achieve a better but limited integration than injections of free cells in the same region, and slightly improve vision capabilities in different experimental models.

To obtain clinically relevant therapeutic solutions, a good effectiveness of the proposed treatment must be achieved. And the key factor to increase the effectiveness of retinal implant therapies is the accomplishment of a substantial integration of the implanted cells. In this aspect an immense amount of work is still needed: the discovery of the exchange of genetic material between native and implanted photoreceptors suggests that the integration of these neurons to the host retina, reported in up-to-date studies, is probably not real, putting on the table the need to verify and reevaluate these studies. It is still unknown whether the therapeutic effect of cell transplants is due to an improvement in functionality by integration or due to production of protective factors. The elucidation of the involved mechanisms is essential for the accomplishment of this goal. Furthermore, functionality analysis of the integrated implants has to be performed.

## Author Contributions

NJ-D and AG-D: bibliographic research and information synthesis, AMD course, wrote the manuscript, and equal contribution. MF-A, NS-B, and NA-L: bibliographic research and information synthesis. FA-M: clinical aspects. GG, JP-R, FR, and DK: biomaterials aspects. DG-N: biomaterials and cell and tissue therapy. GG, JP-R, DK, DG-N: manuscript revision. FP: manuscript design, information synthesis, supervision, and wrote the manuscript. All authors contributed to the article and approved the submitted version.

## Conflict of Interest

The authors declare that the research was conducted in the absence of any commercial or financial relationships that could be construed as a potential conflict of interest.

## Appendix

### Abbreviations List

AMD, Age-Related Macular Degeneration

ARPE-19, human retinal pigment epithelial cell line-19

BrM, Bruch’s membrane

CNTF, ciliary neurotrophic factor

CNV, choroidal neovascularization

D-AMD, Dry Age-Related Macular Degeneration

dRPE, Pluripotent differentiated RPE

ECT, encapsulated cell technology

ELRs, elastin-like recombinamers

ESCs, embryonic- or fetal- derived stem cells

ePTFE, expanded polytetrafluoroethylene

fhRPE, fetal human RPE

FDA, US Food and Drug Administration Agency

GFP, green fluorescent protein

Gnat12/2 mice, model of congenital stationary night blindness

hESCs, human embryonic stem cells

hESCs-RPE cells, human embryonic stem cell-derived RPE cells

hiPSC, human induced pluripotent stem cells

hPSCs-PRs, human pluripotent stem cells-derived photoreceptors

hRPCs, human retinal progenitor cells

hRPE, human RPE

IPE, iris pigment epithelium

IPM, interphotoreceptor matrix

iPSC, induced pluripotent stem cell

mESC, mouse embryonic stem cell

miPSC, mouse induced pluripotent stem cell

MMP2, matrix metallopeptidase 2

mPEG-PLGA, methoxy-poly (ethylene glycol) block-poly lactic-co-glycolic acid

mPEG-PCL, methoxy poly (ethylene glycol) -b-copolymers of polycaprolactone

mSC, mesenchymal stem cell

NP, nanoparticles

NpHR, Natronomonas pharaonis halorhodopsin

PCL, poly(caprolactone)

PDMS, plasma-modified polydimethylsiloxane

PDMS-PmL, plasma-modified polydimethylsiloxane (PDMS) with laminin

PEGDMA, polyethylene glycol dimethacrylate

PEG, poly (ethylene glycol)

PET, polyethylene terephthalate

PGA, polyglycolic acid

PGS, poly(glycerol-sebacate)

PI, polyimide

PLA, poly (DL-lactic acid)

P(LA-co-CL), poly(l-lactide-co-ε-caprolactone)

PLDLA, copolymer 96/4 L-lactide/D-lactide

PLGA, poly(lactic-co-glycolic) acid

PLLA, poly (L-lactic acid)

PLLA/PLGA, poly (L-lactic acid)/poly (lactic-co-glycolic acid)

PMMA, poly (methyl methacrylate)

PPCs, photoreceptor precursor cells

PR, photoreceptors

Prph2+/Δ307, model of retinitis pigmentosa

PSCs, pluripotent stem cells

PTFE, Polytetrafluoroethylene

RGD, arginine–glycine–aspartic acid

RPCs, retinal progenitor cell

RPE, retinal pigment epithelium

RPESC, retinal pigment epithelium stem cells

RSCs, retinal stem cells

SC, stem cell

TDSC, tissue-derived stem cells

TRL, clinical trials

VEGF, vascular endothelium growth factor

VEGF-Fc, VEGF receptor fragment crystallizable region

W-AMD, Wet Age-Related Macular Degeneration
